# The application of exercise stress cardiovascular magnetic resonance in patients with suspected dilated cardiomyopathy

**DOI:** 10.1186/s12968-020-0598-4

**Published:** 2020-02-03

**Authors:** Thu-Thao Le, Jennifer Ann Bryant, Briana Wei Yin Ang, Chee Jian Pua, Boyang Su, Pei Yi Ho, Shiqi Lim, Weiting Huang, Phong Teck Lee, Hak Chiaw Tang, Chee Tang Chin, Boon Yew Tan, Stuart Alexander Cook, Calvin Woon-Loong Chin

**Affiliations:** 1 National Heart Research Institute Singapore, National Heart Center Singapore, 5 Hospital Drive, Singapore, Singapore; 2 Cardiovascular Sciences ACP, Duke NUS Medical School, Singapore, Singapore; 3 Department of Cardiology, National Heart Center Singapore, Singapore, Singapore; 4 National Heart and Lung Institute, Imperial College, London, UK

**Keywords:** Cardiovascular magnetic resonance, Supine bike ergometer, Exercise induced cardiac remodeling, Dilated cardiomyopathy

## Abstract

**Objectives:**

The imaging features of dilated cardiomyopathy (DCM) overlap with physiological exercise-induced cardiac remodeling in active and otherwise healthy individuals. Distinguishing the two conditions is challenging. This study examined the diagnostic and prognostic roles of exercise stress imaging in asymptomatic patients with suspected DCM.

**Methods:**

Exercise stress cardiovascular magnetic resonance (CMR) was performed in 60 asymptomatic patients with suspected DCM (dilated left ventricle and/or impaired systolic function on CMR), who also underwent DNA sequencing for DCM-causing genetic variants. Confirmed DCM was defined as genotype- and phenotype-positive (G+P+). Another 100 healthy subjects were recruited to establish normal exercise capacities (peak exercise cardiac index; Peak_CI_). The primary outcome was a composite of all-cause mortality, cardiac decompensation and ventricular arrhythmic events.

**Results:**

No patients with confirmed G+P+ DCM had Peak_CI_ exceeding the 35th percentile specific for age and sex. Applying this threshold in G-P+ patients, those with Peak_CI_ below 35th percentile had characteristics similar to confirmed DCM while patients with higher Peak_CI_ were younger, more active and higher longitudinal strain. Adverse cardiovascular events occurred only in patients with low exercise capacity (*P* = 0.004).

**Conclusions:**

In individuals with suspected DCM, exercise stress CMR demonstrates diagnostic and prognostic potential in distinguishing between pathological DCM and physiological exercise-induced cardiac remodeling.

## Introduction

Dilated cardiomyopathy (DCM) is the most common cardiomyopathy and a major cause of heart failure [1]. DCM is characterized by left ventricular (LV) dilatation and systolic dysfunction that cannot be explained by coronary artery disease and abnormal loading conditions [2, 3]. A revised definition of DCM was proposed recently to include those with LV dilatation without systolic dysfunction (isolated ventricular dilatation) and hypokinesia without LV dilatation (hypokinetic non-dilated cardiomyopathy) to underscore the heterogeneous phenotypic expression of DCM [2].

In recent studies, LV dilatation and impaired systolic function were reported in physically active healthy subjects using either a self-reported questionnaire or wearable device assessment of activity [4, 5]. Approximately 38% of healthy subjects had a dilated LV on cardiovascular magnetic resonance (CMR) and physical activity was independently associated with more than a two-fold increased risk of LV dilatation [4]. These observations support the notion that exercise-induced cardiac remodeling is a continuum, not only observed in highly trained athletes but also present in active adults who are otherwise healthy.

Distinguishing between physiological exercise-induced cardiac remodeling and DCM has important consequences and long-term life-style effects. Despite the profound clinical implications, current diagnostic approaches that rely on resting parameters lack sufficient accuracy. Molecular genetic analysis of DCM may be helpful but the diagnostic yield is only about 30–35% [6]. Although it has been proposed as a promising modality, the role of exercise stress imaging in patients with suspected DCM is unclear because the diagnostic threshold of contractile reserve has not been well-defined nor validated [7].

We recently developed an exercise stress CMR protocol using a supine ergometer that has been validated against the cardiopulmonary exercise test, the non-invasive gold standard of assessing exercise capacity. Despite similar cardiac index (CI) at rest, competitive athletes had increased CI with exercise compared to healthy sedentary subjects [8]. Extending from our previous work, we hypothesize that exercise stress imaging has potential to distinguish between pathological DCM and physiological exercise-induced cardiac remodeling in patients with suspected DCM. Normal age- and sex-specific peak exercise CI values were first established in healthy subjects. Subsequently, we examined the diagnostic and potential prognostic value of exercise stress CMR in patients with suspected DCM.

## Methods

### Study design and study population

In this single-center prospective outcome study, patients were recruited between April 2016 and August 2018 for exercise stress CMR if they had imaging features of DCM on transthoracic echocardiogram or resting CMR. To be included in this study, patients did not have heart failure (New York Heart Association (NYHA) I) and had to satisfy at least one of the following two criteria using age- and sex-specific Asian CMR references values [2, 9]:

- Dilated LV defined as elevated indexed LV end-diastolic volume (EDV) two standard deviations above normal and/or
- Impaired LV systolic function defined as reduced LV ejection fraction (LVEF)

Patients with other cardiomyopathies such as hypertrophic cardiomyopathy, hypertensive heart disease, arrhythmogenic right ventricular dysplasia, LV non-compaction cardiomyopathy and ischemic cardiomyopathy were excluded. Ischemic cardiomyopathy was defined as the presence of myocardial infarction on CMR or hemodynamically significant coronary artery disease assessed with either invasive or computed tomography coronary angiography, as guided by conventional recommendations [2, 3, 6].

In addition, healthy subjects (*n* = 100) without symptoms, clinical or family history of cardiovascular diseases were prospectively recruited. None of them had any cardiovascular risk factors: hyperlipidemia, diabetes mellitus or hypertension.

The physical activity levels in all participants were evaluated using the established General Practice Physical Activity Questionnaire. The self-administered questionnaire described the type and amount of physical activity in the workplace, the number of hours spent on different activities and their usual walking pace. The activity levels were classified into four categories: inactive, moderately inactive, moderately active and active [10].

### DNA extraction and targeted sequencing of DCM genes

Genetic sequencing was performed in all the patients with suspected DCM. Genomic DNA was extracted from patient’s whole blood using Chemagic DNA blood kit (PerkinElmer, Inc. Waltham, Massachusetts, USA) following the manufacturer’s protocol. Quality and quantity of the extracted DNA were assessed by Labchip Ds (PerkinElmer, Inc), an automated ultraviolet-visible spectrophotometer. Purified DNA was targeted enriched for genes related to DCM using TruSight Cardio kit (Illumina, San Diego, California, USA) according to the manufacturer’s protocol. Pooled libraries were sequenced using Illumina MiSeq (v2 kit) or NextSeq 500 (Mid Output v2 kit) benchtop sequencers using paired-end, 150 bp reads. Raw sequencing data were demultiplexed, trimmed and mapped to UCSC GRCh37/hg19 reference genome as previously described [11]. Variants were called using GATKv3.3 HaplotypeCaller and UnifiedGenotyper annotated using CardioClassifier, a semi-automatic classification on inherited cardiac conditions defined according to the American College of Medical Genetics and Genomics and the Association for Molecular Pathology [12]. The pathogenicity of the variants for each patient was further confirmed by a cardiologist who is an expert in genetics and DCM (SAC). He was blinded to the imaging and other clinical data.

### Cardiovascular magnetic resonance image acquisition

Baseline breath-hold CMR at 1.5 T was performed in all healthy subjects and patients before initiating exercise stress (Siemens MAGNETOM Aera, Siemens Healthineers, Erlangen, Germany). Balanced steady-state free precision (bSSFP) cines were acquired in the standard long-axis (2-, 3- and 4-chamber) and short-axis extending from the base to the apex (8 mm thick and 2 mm gap; TE 1.2 ms, TR 3 ms; field of view 280-320 mm; acquired voxel size 1.6 × 1.3 × 8.0 mm^3^; 30 phases per cardiac cycle).

Exercise stress was performed with a programmable supine ergometer (Lode BV, Groningen, The Netherlands) fitted onto the CMR scanner table. The protocol has been described in detail previously [8]. In brief, the participants cycled with an initial workload of 25 W, at a cadence of at least 70 rpm for 1 min. Workload was gradually increased by 25 W every minute until exhaustion. Real-time prospective ECG-gated bSSFP cines in the short axis were acquired at every stage of the exercise (8 mm thick and 2 mm gap; TE 0.99 ms; TR 2.3 ms; field of view 225 x 300 mm; acquired matrix size 68 × 128 pixels; acceleration factor 4; acquired voxel size 3.3 × 2.3 × 8.0 mm^3^; temporal resolution 39.1 ms). As part of our protocol, patients taking beta-blockers were advised to stop taking them 2 days before the stress test. All healthy subjects and patients with suspected DCM successfully completed the exercise stress CMR protocol and were included in the analysis of this study.

In patients with suspected DCM, replacement and diffuse myocardial fibrosis imaging was assessed using late gadolinium enhanced (LGE) imaging and myocardial T1 mapping, respectively. LGE imaging was performed at 8 min after 0.1 mmol/kg of gadobutrol (Gadovist; Bayer Pharma AG, Germany) was administered. An inversion-recovery fast gradient echo sequence was used, and the inversion time was optimised to achieve appropriate nulling of the myocardium. Myocardial T1 mapping was performed using the Modified Look-Locker Inversion-recovery (MOLLI) sequence (flip angle 35; minimum TI 100 ms; TI increment of 80 ms). Native and 15-min post-contrast T1 maps were acquired using a heartbeat acquisition scheme of 5(3)3 and 4(1)3(1)2, respectively.

### Cardiovascular magnetic resonance image analysis

All image analysis was performed using standardized protocols at our **NHRIS CMR Core Laboratory** (CVi42, Circle Cardiovascular Imaging, Calgary, Canada). LV mass and cardiac volumes at baseline (breath-hold short axis cine images) and each exercise stage (real-time short axis cine images) were assessed in all patients. The exercise stress protocol demonstrated excellent scan-rescan (0.2 ± 05 L/min/m^2^) and inter-observer (LVEDV: 2.8 ± 5.2 mL; LVESV: − 0.5 ± 5.2 mL) reproducibility [8]. Exercise-related parameters examined were relative change in exercise LVEF, relative change in exercise CI and peak exercise CI expressed as age- and sex-specific percentiles.

Myocardial strain was analysed in the breath-hold cine images using the Tissue Tracking Plugin. Circumferential and radial strain were measured in the short axis cine images, whilst longitudinal strain was measured in the three long-axis views [13]. The amount of replacement fibrosis was quantified using a signal intensity threshold greater than two (2-SD) and five times (5-SD) the standard deviation above the mean value in a normal region of myocardium sampled in the same short-axis slice. Areas of artefacts or contamination (blood pool and/or epicardial fat) were manually excluded. The higher threshold was accurate compared with histological fibrosis whilst the lower threshold predicted adverse prognosis in patients with DCM [14, 15]. Diffuse myocardial fibrosis was assessed using extracellular volume fraction (ECV), estimated from the native and 15-min post contrast T1 map [16]. Interstitial volumes were defined as a product of ECV and myocardial volume (myocardial volume = LV mass/1.05 g/mL), a measure that correlated very well with histological fibrosis (*r* = 0.87, *P* < 0.001) and had prognostic value in patients with aortic stenosis [17].

### Study outcomes

Available clinical outcome data in all patients with suspected DCM were reviewed in June 2019. The primary outcome was a composite of all-cause mortality, cardiac decompensation and ventricular arrhythmias. Cardiac decompensation was defined as objective evidence of decreased LVEF and elevated brain natriuretic peptides, corroborated with heart failure symptoms; and ventricular arrhythmias were defined as sudden cardiac deaths, ventricular tachyarrhythmias (sustained and non-sustained) and ventricular fibrillation. These are clinically relevant end-points in DCM that were carefully adjudicated by an experienced cardiologist (CWLC), who was blinded to the imaging, genetics and clinical data.

### Statistical analysis

Distribution of continuous variables was assessed using the Shapiro-Wilk test. Data were presented in either mean ± standard deviation or median [inter-quartile range], as appropriate. Depending on the normality of the distribution, parametric Student’s t test and 1-way ANOVA or the nonparametric Mann-Whitney U test and Kruskal-Wallis test were used to compare groups of continuous data. Categorical data were compared using the χ^2^ test. The prognostic association of the peak exercise CI threshold was assessed using the Kaplan-Meier analysis. Statistical analyses were performed using GraphPad Prism 8.0.1 (GraphPad Software, Inc., San Diego, California, USA) and SPSS Version 24 (Statistical Package for the Social Sciences (SSPS), International Business Machines, Inc., Armonk, New York, USA). A two-sided *P*-value <0.05 was considered as statistically significant.

## Results

### Normal exercise capacity ranges established in healthy subjects

**Fig. 1:**
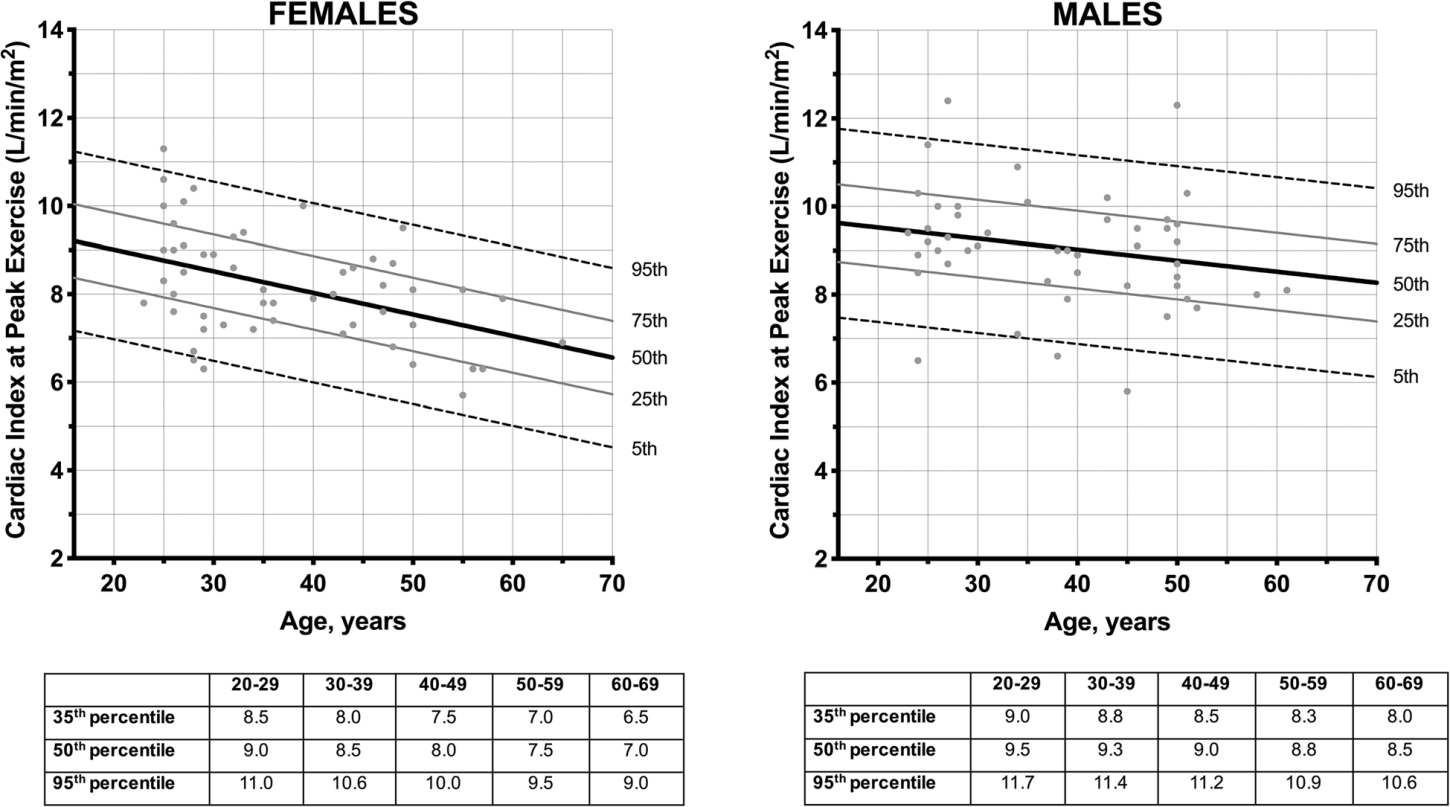
Distribution of Peak Exercise Cardiac Index in Healthy Subjects

Most of the healthy subjects (76%) were at least moderately active. Cardiac volumes, LV mass and systolic function (including myocardial strain measures) were within normal limits (Table 1). Peak exercise CI was higher in males than females (9.0±1.3 versus 8.2±1.2 L/min/m^2^; *P* < 0.001) and correlated negatively with age (*r* = − 0.30; *P* < 0.001). There were no associations between peak exercise CI and intensity of physical activity on self-administered questionnaire, body mass index, heart rate or blood pressures (*P* > 0.05). Reference ranges in peak exercise CI were established in the healthy subjects, stratified by sex (Fig. 1). The peak exercise heart rate achieved was 149 [137 to 160] beats per min, corresponding to 82 [76 to 86] % of the age-predicted maximal heart rate. All achieved at least 50% of the age-predicted maximal heart rate.

**Table 1:** Characteristics of Healthy Subjects and Patients with Suspected Dilated Cardiomyopathy

	Healthy Subjects (*n* = 100)	Patients with suspected DCM (*n* = 60)	*P* Value
CLINICAL CHARACTERISTICS			
Age, years	38 ± 11	34 ± 14	0.102
Males, n (%)	48 (48)	56 (93)	<0.001
BSA, m^2^	1.71 ± 0.22	1.92 ± 0.23	<0.001
BMI, kg/m^2^	22.7 ± 3.3	26.1 ± 5.8	<0.001
Physical Activity			
Inactive, n (%)	13 (13)	14 (23)	0.406
Moderately Inactive, n (%)	11 (11)	7 (12)	
Moderately Active, n (%)	17 (17)	9 (15)	
Active, n (%)	59 (59)	30 (50)	
Heart Rate, bpm	70 ± 13	68 ± 15	0.342
Systolic Blood Pressure, mmHg	120 ± 15	122 ± 15	0.517
Diastolic Blood Pressure, mmHg	72 ± 11	72 ± 14	0.703
CMR CHARACTERISTICS			
Indexed LV mass, g/m^2^	47 ± 9	66 ± 13	<0.001
Maximal Wall Thickness, mm	7.5 ± 1.2	8.8 ± 1.6	<0.001
Indexed LV EDV, mL/m^2^	80 ± 11	115 ± 20	<0.001
Indexed LV ESV, mL/m^2^	33 ± 7	63 ± 19	<0.001
LV Ejection Fraction, %	59 ± 5	45 ± 9	<0.001
Indexed RV EDV, mL/m^2^	89 ± 13	109 ± 26	<0.001
Indexed RV ESV, mL/m^2^	42 ± 10	58 ± 16	<0.001
RV Ejection Fraction, %	53 ± 6	47 ± 7	<0.001
Circumferential Strain, %	−23.3 ± 2.7	−16.0 ± 3.7	<0.001
Radial Strain, %	50.6 ± 10.1	28.1 ± 9.5	<0.001
Longitudinal Strain, %	−20.9 ± 2.4	−16.4 ± 3.6	<0.001

Abbreviations: *BSA* Body surface area, *BMI* Body mass index, *LV* Left ventricular, *RV* Right ventricular, *DCM* Dilated cardiomyopathy, *EDV* end-diastolic volume, *ESV* end-systolic volume

### Diagnostic value of the exercise CMR in patients with suspected DCM

A total of 60 asymptomatic patients with suspected DCM were recruited (Table 1). Most of them were at least moderately active based on the self-administered questionnaire. We defined confirmed DCM on the basis of positive genotypic and phenotypic (G+P+) expressions. There were 13 (21%) patients with confirmed DCM: 92% of pathogenic gene mutations were in the TTN gene (premature stop, frameshift and essential splice: I-band, *n* = 3; A-band, *n* = 9) and one missense in MYH7. The clinical and CMR characteristics were presented in Table 2. In the patients with confirmed DCM, 15% had a positive family history of cardiomyopathy and 38% had non-ischemic replacement fibrosis on CMR.

**Table 2:** Characteristics of Patients Suspected Dilated Cardiomyopathy Stratified by Genetic Status and Exercise Capacity

	Confirmed DCM (Pathogenic Gene Mutation) (*n* = 13)	No Gene Mutation			
		Low Exercise Capacity (*n* = 16)	*P* Value^Π^	High Exercise Capacity (*n* = 31)	*P* Value^Π^
CLINICAL CHARACTERISTICS					
Age, years	43 [22,50]	43 [39,48]	0.746	22 [19,39]	0.033
Males, n (%)	11 (85)	14 (88)	1.000	31 (100)	0.082
BSA, m^2^	1.86 [1.68,2.00]	1.99 [1.88,2.17]	0.075	1.92 [1.79,2.02]	0.537
BMI, kg/m^2^	24.9 [21.8,28.1]	30.4 [24.2,35.4]	0.132	24.1 [20.6,27.3]	0.580
Physical Activity					
Inactive, n (%)	4 (31)	6 (38)	0.434	1 (3)	0.339
Moderately Inactive, n (%)	2 (15)	4 (25)		1 (3)	
Moderately Active, n (%)	2 (15)	4 (25)		3 (10)	
Active, n (%)	5 (39)	2 (12)		26 (84)	
Heart Rate, bpm	73 [66,79]	78 [68,90]	0.308	60 [53,68]	0.004
Peak Exercise Heart Rate, bpm	140 [120,152]	134 [119,149]	0.650	160 [147,165]	0.002
Age-predicted Maximal Heart Rate, %	74 [66,87]	76 [64,81]	0.846	82 [75,87]	0.168
Systolic Blood Pressure, mmHg	116 [101,136]	120 [112,133]	0.329	126 [118,131]	0.102
Diastolic Blood Pressure, mmHg	76 [64,84]	81 [69,85]	0.475	65 [58,79]	0.149
Hypertension, n (%)	3 (23)	7 (44)	0.433	3 (10)	0.339
Diabetes, n (%)	3 (23)	5 (31)	0.697	1 (3)	0.071
Fam. Hist. of Cardiomyopathy, n (%)	2 (15)	0	0.192	0	0.082
Fam. Hist. of Sudden Cardiac Death, n (%)	0	2 (13)	0.488	0	1.000
CMR CHARACTERISTICS					
Indexed LV mass, g/m^2^	54 [45,63]	69 [55,82]	0.009	70 [62,76]	<0.001
Indexed LV EDV, mL/m^2^	102 [91,127]	106 [88,127]	0.914	118 [110,130]	0.021
Indexed LV ESV, mL/m^2^	59 [48,88]	65 [49,81]	0.914	57 [50,71]	0.671
LV Ejection Fraction, %	42 [32,47]	41 [34,48]	0.880	51 [46,55]	<0.001
Indexed RV EDV, mL/m^2^	81 [74,106]	89 [76,112]	0.449	125 [112,136]	<0.001
Indexed RV ESV, mL/m^2^	45 [36,64]	46 [41,63]	0.589	64 [55,71]	0.005
RV Ejection Fraction, %	49 [39,53]	46 [43,48]	0.880	48 [45,51]	0.633
Late gadolinium enhancement (LGE), n (%)	5 (38)	5 (31)	0.714	5 (16)	0.131
Amount of LGE (2-SD threshold), %	17.1 [11.5,30.8]	17.1 [11.5,30.8]	1.000	10.0 [7.9,19.7]	0.222
Amount of LGE (5-SD threshold), %	4.1 [2.1,14.3]	4.1 [3.0,10.0]	1.000	4.1 [1.8,5.7]	0.690
Native T1, ms	1047 [1029–1033]	1050 [963–1077]	1.000	987 [977–1033]	0.231
Extracellular volume fraction, %	29.2 [27.4,30.9]	24.7 [21.7,27.8]	0.286	22.1 [20.3,29.3]	0.308
Indexed interstitial volume, mL/m^2^	14.4 [10.1,18.6]	17.1 [14.8,19.7]	0.643	16.0 [14.0,21.1]	0.641
Circumferential Strain, %	−13.3 [−11.3,-16.5]	−12.9 [−11.3,-17.5]	0.507	−17.9 [−15.7,-19.7]	<0.001
Radial Strain, %	24.2 [16.7,27.1]	18.9 [17.4,28.6]	0.838	32.2 [28.1,38.0]	<0.001
Longitudinal Strain, %	−15.9 [−14.6,-16.2]	−14.2 [− 12.3,-16.1]	0.474	− 17.8 [−16.6,-19.8]	0.001
Change in Exercise LVEF, %	49 [34,64]	34 [13,49]	0.036	45 [36,51]	0.463
CI at Rest, L/min/m^2^	2.8 [2.3,3.2]	3.1 [2.9,3.3]	0.062	3.4 [3.0,3.9]	0.001
CI at Peak Exercise, L/min/m^2^	6.9 [5.8,8.3]	7.0 [6.2,7.6]	0.948	11.4 [10.3,12.7]	<0.001
Change in Exercise CI, %	145.8 [101.8182.1]	123.8 [87.5160.3]	0.423	221 [181.6261.3]	<0.001

^Π^compared to confirmed DCM (Pathogenic Gene Mutation)

Abbreviations: *CI* Cardiac index

**Fig. 2:**
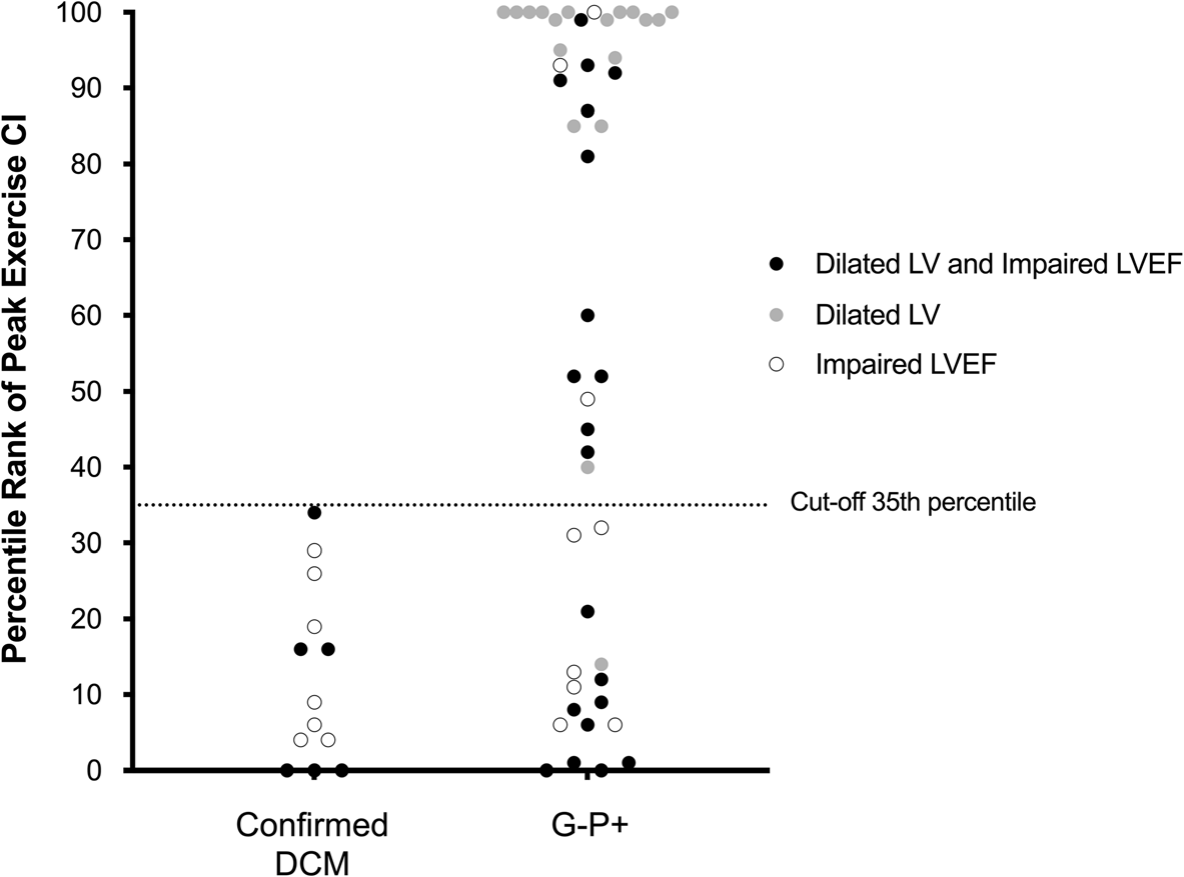
Peak Exercise Cardiac Index of Patients with Suspected Cardiomyopathy Stratified by Genotype. No patient with confirmed DCM had a peak exercise CI exceeding 35th percentile

No patients with confirmed DCM had a peak exercise CI more than 35th percentile specific for age and sex (Fig. 2). Applying this threshold in G-P+ patients (*n* = 47), those patients with peak exercise CI less than 35th percentile (*n* = 16; 34%) shared similar characteristics as confirmed DCM in terms of demographic profiles, activity levels and myocardial strain values (Table 2). Of the 29 patients with low peak exercise CI, most did not have pathogenic gene mutation (*n* = 16; 55%) or fibrosis on CMR (*n* = 19; 66%). Approximately half had only one abnormal phenotype (13 had impaired LVEF and one had a dilated LV). Conversely, G-P+ patients with peak exercise CI greater than 35th percentile (*n* = 31; 66%) were younger, more active, had enlarged right ventricles and increased myocardial strain values compared to confirmed DCM (Table 2). Of the 31 patients, 87% and 80% of them were able to achieve peak CI exceeding 50th percentile and 80th percentile, respectively. Of note, the change in exercise LVEF and the extent of non-ischemic fibrosis were not able to distinguish between confirmed DCM and those with high exercise capacity (Table 2).

### Prognostic value of exercise CMR in patients with suspected DCM

Over 21 ± 9 months of follow-up, there were 7 events (event rate of 6.0 events/100 patient-years). All seven patients developed evidence of cardiac decompensation during follow-up: LVEF decreased by 10–29% and NT-proBNP concentrations were elevated between 973 and 6701 pg/mL (all patients had normal renal function). One patient underwent implantation for LV assist device, one was advised for cardiac resynchronization therapy with defibrillation, and the third patient had an arrhythmic event. This patient developed episodes of non-sustained ventricular tachycardia, the longest was 7 s that occurred during sleep. She eventually received an implantable cardioverter-defibrillator. There were no deaths. In the 7 patients with adverse events, one in two had a pathogenic gene mutation and/or non-ischemic type replacement fibrosis on CMR (Additional file 1).

**Fig. 3:**
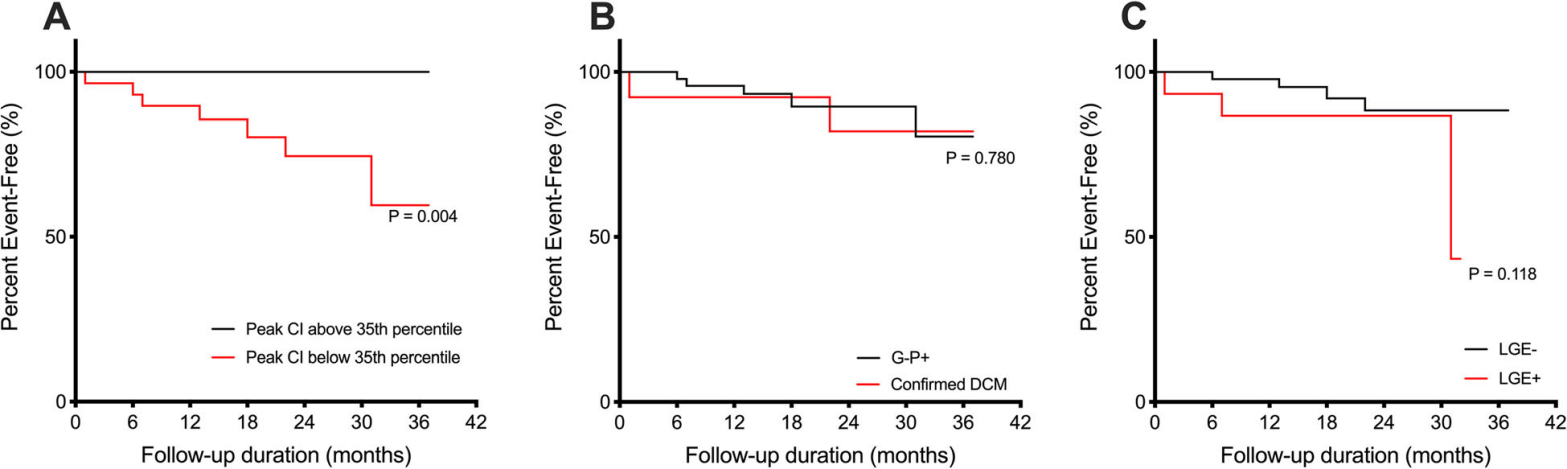
Outcome Analysis of Patients with Suspected Cardiomyopathy. Individuals with low peak exercise cardiac index (CI) less than 35th percentile for age and sex and worse prognosis compared to those with higher peak exercise CI **(a**). There was no difference in prognosis stratified by either genotype status (**b**) or fibrosis status on cardiovascular magnetic resonance (**c**)

Despite small numbers and limited follow-up, we found prognostic significance of exercise CMR: all 7 events occurred in patients with low exercise capacities whilst those with peak exercise CI more than 35th percentile had no events (*P* = 0.004; Fig. 3). Of importance, there was no difference in event distribution stratified by either genotype status (*P* = 0.780) or fibrosis status on CMR (*P* = 0.118; Fig. 3). The other exercise-related parameters (heart rate and systolic blood pressure) were similar between those with and without adverse events (Additional file 1).

## Discussion

Here for the first time, the diagnostic value and prognostic implications of exercise stress imaging were demonstrated in patients with suspected DCM. Based on our inclusion and exclusion criteria, these asymptomatic patients with suspected DCM could have either early pathological DCM or normal physiological cardiac remodeling because of their active lifestyles. Using the exercise capacities in healthy subjects and patients with confirmed DCM, a peak exercise CI less than 35th percentile was defined as low exercise capacity in this study. Patients with suspected DCM and low peak exercise CI experienced early adverse outcomes. Conversely, patients with higher exercise capacities had excellent prognosis and did not have any adverse cardiovascular events during follow-up. These findings support the role of exercise stress imaging in patients with suspected DCM.

Cardiac imaging is the fundamental tool of establishing the etiology of cardiomyopathies. However, distinguishing DCM from cardiac remodeling in athletes and active healthy subjects can be challenging given the overlapping imaging features at rest. Exercise stress coupled with imaging has theoretical advantage to rest-based imaging. Although echocardiography remains the mainstay of cardiac imaging, it has limitations in distinguishing overlapping phenotypes between DCM and exercise-induced cardiac remodeling. CMR is widely considered the gold standard for the assessment of cardiac function and LV mass. Moreover, the strength of myocardial characterization with gadolinium contrast in CMR is unparalleled in other conventional imaging modalities [18]. For these reasons, we used the recently developed exercise stress CMR to examine the role of exercise stress imaging in DCM [8].

In addition to comprehensive phenotyping with CMR, all patients with suspected DCM underwent targeted sequencing and careful variant annotation. Confirmed DCM was defined on the basis of both phenotypic and genotypic expression (G+P+). We then examined how exercise CMR was able to distinguish between pathological DCM and exercise-induced cardiac remodeling in the group of G-P+ patients. More than 30% of them had low exercise capacities and their characteristics overlapped with confirmed G+P+ DCM that support the diagnosis of pathological DCM even in the absence of pathogenic gene mutation. Moreover, low peak exercise CI was associated with a worse prognosis. Conversely, G-P+ patients with high peak exercise CI were younger, significantly more active based on self-reported questionnaire and increased strain values compared to confirmed G+P+ DCM. No patients with high peak exercise CI more than 35th percentile experienced any adverse outcomes during follow-up. This would suggest that dilated LV and/or impaired LV function in these individuals were features of physiological cardiac remodeling from their active lifestyles, despite not being in competitive sports.

Amongst those with low peak exercise CI (and thus likely have pathological DCM), 55% of them did not have a known pathogenic gene mutation, in keeping with expected diagnostic yield of molecular genetic testing in DCM. More than 65% of them did not have fibrosis on CMR. Given that rest-based imaging markers such as myocardial fibrosis and longitudinal strain predict worse outcomes in DCM patients with longer follow-up [18–22], it is possible that the predictive value of exercise CMR is equally or more informative. The role of exercise stress CMR should be confirmed in large populations with longer follow-up duration in order to elucidate how exercise stress imaging can be integrated with other molecular and imaging prognostic markers.

### Clinical implications

CMR has a well-established clinical role in ascertaining the underlying etiology in DCM [23]. Unlike cardiopulmonary exercise test or echocardiography, exercise stress CMR can assess cardiac function, myocardial mass, tissue characterization and exercise capacities in a single modality. Unlike exercise LVEF and other imaging parameters, peak exercise CI is a measure that incorporates a physiologic parameter (heart rate) in the assessment of exercise capacity. This could partially explain the ability of peak exercise CI to distinguish between pathological DCM and physiological exercise-induced cardiac remodeling.

DCM remains a challenging diagnosis in some patients despite advances in cardiac imaging. Particularly in patients with imaging features of DCM and do not have a pathogenic gene mutation, exercise stress CMR may increase the diagnostic confidence of distinguishing between pathological DCM and physiological exercise-induced cardiac remodeling. Further investigations are needed to discern the role of exercise stress imaging in differentiating between physiology and pathology in highly trained athletes with increased cardiac volumes and mildly impaired LVEF [24].

### Study limitations

The event rates were low because of the short follow-up duration and the patients were asymptomatic (NYHA I) at the time of recruitment, which precluded further analysis to examine the incremental and independent prognostic value of the exercise stress imaging compared to other established prognostic markers. Despite these limitations, we observed that only patients with low peak exercise CI developed adverse prognosis. In the absence of any well-validated diagnostic criteria of DCM, we had used very stringent definition of confirmed DCM (positive genotype and phenotype). As a consequence, the sample size is relatively small, but an irrefutable diagnosis of DCM is essential to test the potential of low peak exercise CI. Most of the patients with suspected DCM were males. However, the peak exercise CI was expressed as age- and sex-specific percentiles to minimize any impact (if any) on the overall validity of the study. As echocardiography was not performed in the study, the diagnostic and prognostic value of echocardiographic parameters such as global longitudinal strain and diastolic function cannot be compared with peak exercise CI.

## Conclusion

Pathological DCM and physiological cardiac adaptation from active lifestyles have overlapping rest-based imaging features. Exercise stress CMR has potential diagnostic and prognostic value in distinguishing between pathological DCM and physiological exercise-induced cardiac remodeling in patients with suspected DCM.

## Supplementary information

**Additional file 1.** Clinical and CMR characteristics between patients with and without adverse events; Exercise parameters between patients with and without adverse events.

## Data Availability

The datasets generated and analysed during the current study are not publicly available. Please contact the corresponding author for data requests.

## Abbreviations

bSSFP: Balanced steady-state free precision
CI: Cardiac index
CMR: Cardiovascular magnetic resonance
DCM: Dilated cardiomyopathy
ECV: Extracellular volume fraction
EDV: End-diastolic volume
LGE: Late gadolinium enhancement
LV: Left ventricular/left ventricle
LVEDV: Left ventricular end-diastolic volume
LVEF: Left ventricular ejection fraction
LVESV: Left ventricular end-systolic volume
MOLLI: Modified Look-Locker inversion recovery
NYHA: New York Heart Association
