# Supplementary material for: The application of exercise stress cardiovascular magnetic resonance in patients with suspected dilated cardiomyopathy
Source: J Cardiovasc Magn Reson. 2020 Feb 3;22:10. doi: 10.1186/s12968-020-0598-4 (PMC6996168; doi:10.1186/s12968-020-0598-4)
Supplement: Supplementary file 1 — Additional file 1. Clinical and CMR characteristics between patients with and without adverse events; Exercise parameters between patients with and without adverse events. [file 12968_2020_598_MOESM1_ESM.docx]

**The Application of Exercise Stress Cardiovascular Magnetic Resonance in Patients with Suspected Dilated Cardiomyopathy**

**ONLINE SUPPLEMENTAL DATA**

Clinical and CMR characteristics were similar between patients with and without adverse events (except more males in those without events).

**Characteristics of low exercise response patients with and without adverse events.**

|  | **Events**  (n = 7) | **No Events**  (n = 22) | **P value** |
| --- | --- | --- | --- |
| **CLINICAL CHARACTERISTICS** | | | |
| Age, years | 41 [23-49] | 44 [36-48] | 0.746 |
| Males, n (%) | 4 (57) | 21 (95) | **0.034** |
| NYHA, n (%)  I  II  III  IV | 6 (86)  1 (14)  0 (0)  0 (0) | 20 (91)  2 (9)  0 (0)  0(0) | 1.000 |
| Hypertension, n (%) | 3 (43) | 7 (32) | 0.665 |
| Diabetes, n (%) | 2 (29) | 6 (27) | 1.000 |
| Fam. Hist. Cardiomyopathy, n (%) | 1 (14) | 1 (5) | 0.431 |
| Fam. Hist. Sudden Death, n (%) | 2 (29) | 0 (0) | 0.052 |
| Peak Exercise Heart Rate, bpm | 131 [124-162] | 139 [115-150] | 0.650 |
| Age-predicted Maximal Heart Rate, % | 77 [63-82] | 75 [65-83] | 0.846 |
| Peak Exercise Systolic Blood Pressure, mmHg | 161[125-198] | 177 [156-197] | 0.566 |
| Pathogenic gene mutation, n (%) | 2 (29) | 11 (50) | 0.410 |
| **CMR CHARACTERISTICS** | | | |
| Late gadolinium enhancement (LGE), n (%) | 3 (43) | 7 (32) | 0.665 |
| Amount of LGE (2-SD threshold), % | 4.1 [4.0-14.8] | 4.0 [2.0-13.8] | 1.000 |
| Amount of LGE (5-SD threshold), % | 19.6 [15.0-33.7] | 14.7 [13.3-28.0] | 1.000 |

**Breakdown of Events by Genotype Status**

| **Outcomes** | **G+P+**  **(n = 2)** | **G-P+**  **(n = 5)** |
| --- | --- | --- |
| LV assist device implantation | 1 | 0 |
| ICD implantation for ventricular arrhythmias | 1 | 0 |
| Heart failure | 0 | 5 |

Exercise parameters (heart rate and systolic blood pressure) were similar between those with and without adverse events

**
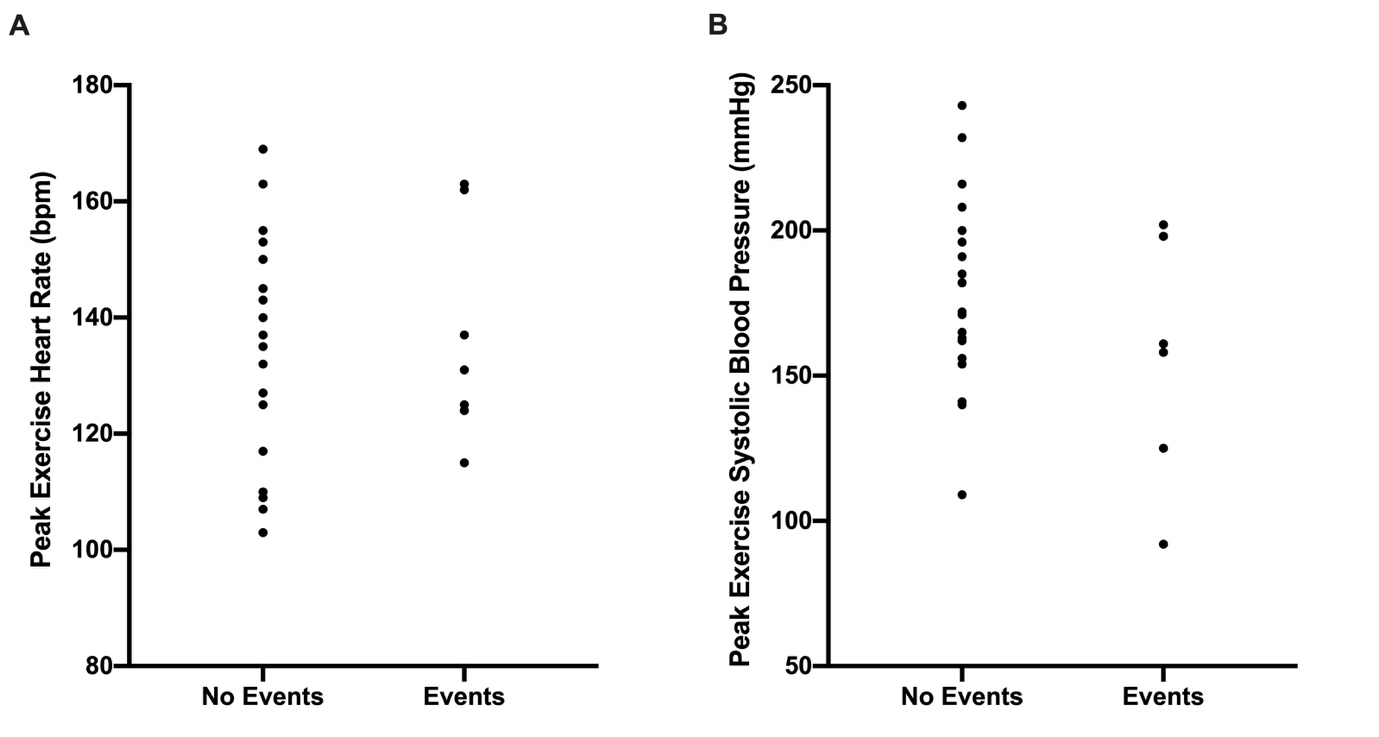
Figure. (A) Peak exercise heart rate and (B) peak exercise systolic pressure of low exercise response patients with and without adverse events**
